# Tracheal neurofibroma treated by repeated flexible bronchoscopic cryotherapy: A case report

**DOI:** 10.1097/MD.0000000000029739

**Published:** 2022-06-30

**Authors:** Ji Soo Yoo, Yeong Hun Choe, Yong Chul Lee, So Ri Kim

**Affiliations:** a Department of Chest Surgery, Jeonbuk National University Medical School, Jeonju-si, South Korea; b Division of Respiratory Medicine and Allergy, Department of Internal Medicine, Jeonbuk National University Medical School, Jeonju-si, South Korea; c Research Institute of Clinical Medicine of Jeonbuk National University, Biomedical Research Institute of Jeonbuk National University Hospital, Deokjin-gu, Jeonju-si, South Korea.

**Keywords:** bronchoscopic cryotherapy, dyspnea, neurofibroma, trachea

## Abstract

Isolated endotracheal neurofibroma is very rare. The majority of neurofibromas are benign, but rarely, they can become cancerous. Furthermore, symptomatic neurofibroma is usually treated by surgical resection. Recently, several alternative options for surgical resection of this airway pathology have been suggested and developed, including bronchoscopic cryotherapy. Nevertheless, there has been no report on the application of repeated flexible bronchoscopic cryotherapy to remove an endotracheal neurofibroma.

**Patient concerns:**

A 65-year-old woman presented with progressive dyspnea and productive cough. Chest computed tomography scans revealed a 1.5-cm polypoid-shaped mass with fat attenuation and mild enhancement in the distal trachea. Flexible bronchoscopic cryotherapy was performed to remove the mass and confirm the diagnosis.

**Diagnosis:**

Pathologically, the mass was diagnosed as an endotracheal neurofibroma occupying the distal tracheal lumen.

**Interventions:**

The endotracheal neurofibroma was completely removed with repeated flexible bronchoscopic cryotherapy instead of surgical resection.

**Outcomes:**

Follow-up flexible bronchoscopy also revealed that there was no regrowth of the neurofibroma. Up to 18 months after the completion of serial cryotherapy, the patient had no recurrent symptoms or complaints.

**Lessons:**

Flexible bronchoscopic cryotherapy can be performed repeatedly for therapeutic purposes for airway tumors. It is recommended to consider flexible bronchoscopic cryotherapy as an alternative therapeutic option for patients with central airway obstruction due to tumorous lesions such as neurofibromas.

## 1. Introduction

Neurofibroma is a benign nerve sheath tumor usually located in the posterior mediastinum that originates from a proliferation of Schwann cells, fibroblasts, perineural cells, and mast cells.^[1]^ Neurofibroma is generally associated with neurofibromatosis type I; however, solitary neurofibromas can develop. The majority of neurofibromas are benign, and rarely, they can become cancerous. It has been reported that up to approximately 10% of neurofibromas may undergo malignant change.^[2]^ In terms of treatment, neurofibromas usually need no treatment for a single, small (i.e., less than approximately 2 cm) tumor in the skin. However, in the case of neurofibromas developing in particular organs, symptoms can be caused by the mass pressing on nearby tissues or by damaging organs. Although the type of surgery depends on the location of neurofibroma, surgical resection has been accepted as the standard treatment for symptomatic neurofibromas.

Cryotherapy is a semi-invasive therapeutic tool for treatment of various pathologic lesions; it uses a cryoprobe at freezing or near-freezing temperatures. The principle of cryotherapy is that when a variety of pathologic lesions are exposed to very low temperatures, the tissues are frozen quickly at the same time and are fatally injured, resulting in cellular damage and local destruction of the target lesions.^[3]^ Cryotherapy causes the lesions to become dehydrated and damaged by deep-freezing-induced vascular injuries, including platelet aggregation, high blood viscosity, vasoconstriction, formation of microthrombi, and tissue ischemia.^[4]^ Based on this principle, cryotherapy has been used to freeze off cancer cells and to remove several types of neoplasms.

In 1968, the first bronchoscopic cryotherapy was performed on an endobronchial tumor by Gage et al.^[5]^ Since then, bronchoscopic cryotherapy has frequently been used to relieve endobronchial obstruction by extracting foreign bodies and reducing or removing malignant tumors. Despite the fact that bronchoscopic cryotherapy has been widely available and the related techniques and devices have been developed, currently, its use on benign masses of the central airway is unfamiliar to the majority of physicians.^[6]^ Moreover, the application of flexible bronchoscopic cryotherapy to treat tracheal obstructing masses is extremely rare.

Here, we present an unusual case of a 65-year-old woman who reported shortness of breath due to an endotracheal neurofibroma; she was successfully treated with repeated flexible bronchoscopic cryotherapy.

## 2. Case report

**Figure 1.**
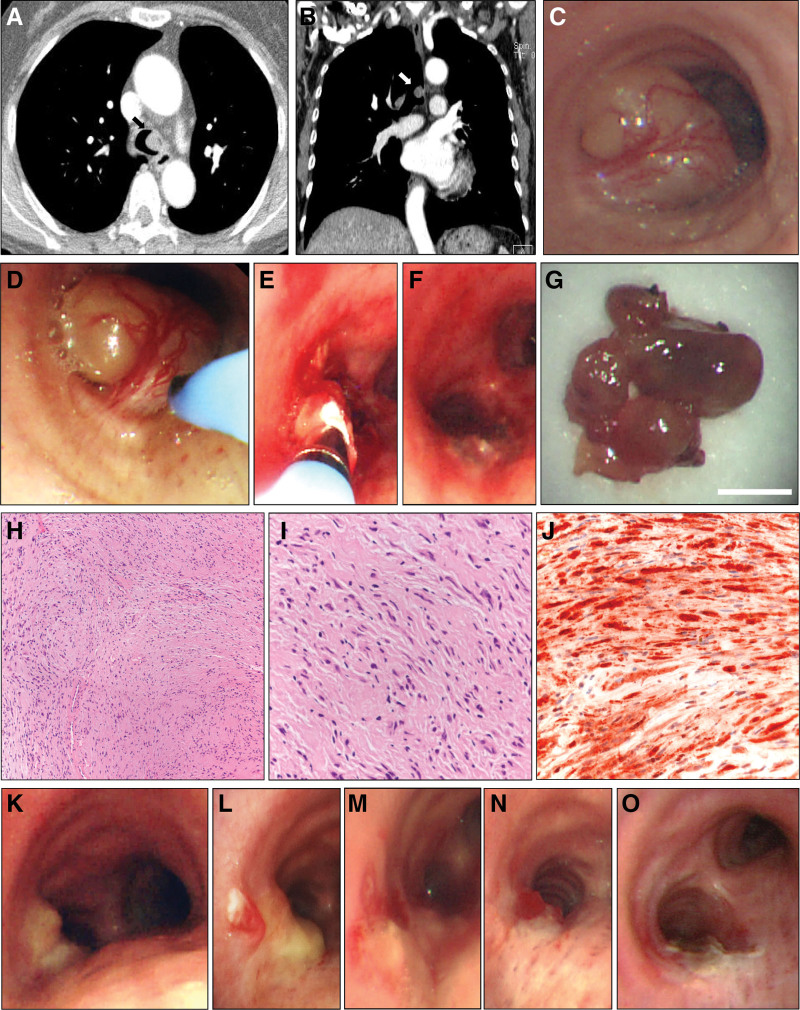
The endotracheal neurofibroma. (A) Transverse view of the chest CT scan. (B) Coronal view of the chest CT scan. Arrows indicate the endotracheal neurofibroma. (C) Bronchoscopic findings of the mass located on the left posterolateral wall. (D–F) Images of extraction of the mass using a cryoprobe with bronchoscopy. (G) Gross image of the tissues removed and dissected by the cryoprobe. White scale bar represents 5 mm. (H) H&E-stained tissues showing the loose arrangement of cells at low magnification (×100). (I) H&E-stained tissues showing spindle-shaped cells with tapered nuclei at high magnification (×400). (J) The positive reaction of the tissues to S-100 protein immunohistochemistry staining at high magnification (×400). (K–O) Serial flexible bronchoscopic cryotherapy. Serial bronchoscopic procedures were performed at a 1-wk interval. K is the picture from the initial bronchoscopic procedure. The alphabetical order of the label in figures is the order of time. CT = computed tomography, H&E = hematoxylin and eosin.

A 65-year-old woman was admitted to a tertiary hospital due to dyspnea of modified Medical Research Council Dyspnea Scale grade 2 and productive cough. According to her history, she had undergone choledochojejunostomy for a choledochal cyst type I 13 years ago. She also had been taking antihypertensive medications for 3 years. She was a nonsmoker and a social drinker. She had no specific family history. Physical examination revealed stridor at the substernal area, but no other remarkable findings such as skin lesions were observed. Pulmonary function test data revealed a forced vital capacity of 2.92 L (111% predicted), forced expiratory volume in 1 second of 2.16 L (114% predicted), forced expiratory volume in 1 second/forced vital capacity of 74%, and no bronchodilator response. Chest computed tomography scans revealed a 1.5-cm polypoid-shaped mass with fat attenuation and mild enhancement in the distal trachea (Fig. 1A, B). On bronchoscopy, the mass was located on the left posterolateral wall of the trachea, approximately 1 cm above the carina. It was well capsulated, demarcated, and hypervascularized (Fig. 1C).

Flexible bronchoscopic cryotherapy was performed to remove the mass and confirm the diagnosis (Fig. 1D–F). Gross examination revealed a gray-colored, glossy, mass-like lesion (Fig. 1G). Microscopically, the mass had loose arrangements of spindle-shaped cells with tapered nuclei (Fig. 1H, I). The cells showed strong diffuse immunoreactivity for S-100 protein (Fig. 1J). Staining tests for cytokeratin, actin, desmin, CD34, and anaplastic lymphoma kinase were all negative. These pathologic findings supported the diagnosis of neurofibroma. Additional systemic examination revealed that there were no other pathologic lesions related to the endotracheal neurofibroma. Therefore, the patient was diagnosed as having an isolated endotracheal neurofibroma.

We recommended surgical resection or rigid bronchoscopic removal as the standard therapeutic option. The patient rejected surgical treatment and rigid bronchoscopy due to the fear of general anesthesia. Therefore, we decided to perform serial flexible bronchoscopic cryotherapies to remove remnants of the neurofibroma. A total of 6 flexible bronchoscopies were done at a 1-week interval. When each intervention was finished, the procedure site was observed to be covered by exudate and necrotic debris. As the procedure was repeated, the amount of exudate and necrotic debris decreased, and the cryotherapy-induced damaged lesions changed into fibrotic tissues (Fig. 1K–O). The patient had no complaints during the repeated procedures, and no clinically significant complications were found.

**Figure 2.**
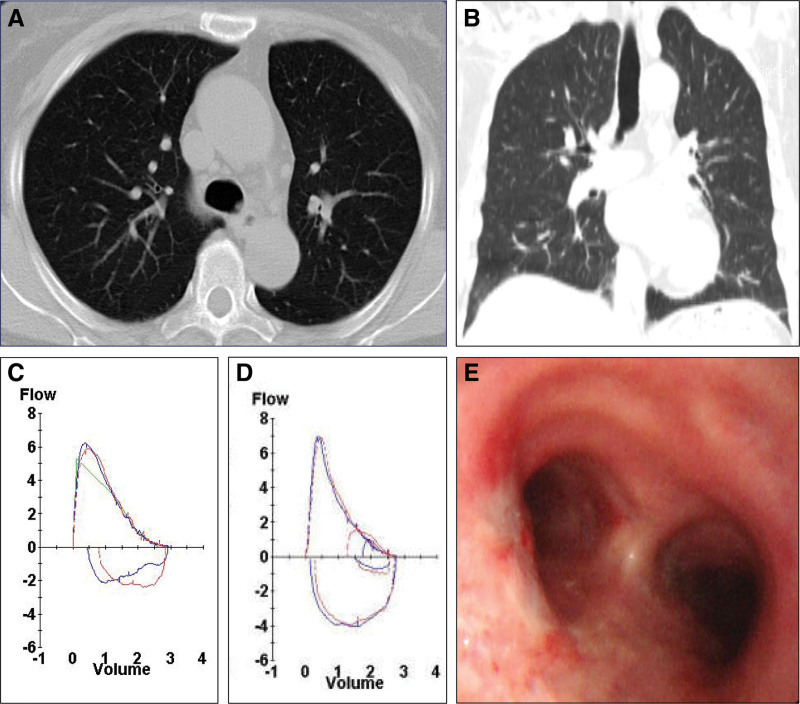
Follow-up examinations 1 mo after the completion of the treatment. Chest CT scans of the transverse (A) and coronal views (B). Flow-volume curves from the initial (C) and follow-up (D) pulmonary function tests. (E) The bronchoscopic picture showing the healing stage with fibrotic scar formation on the previous site of the endotracheal neurofibroma. CT = computed tomography.

Follow-up examinations were performed 1 month after the completion of the final intervention. There was no evidence of recurrence of neurofibroma on computed tomography scans (Fig. 2A, B). On pulmonary function testing, the flow-volume curve showed improvement in the inspiratory flow curve compared to that of the initial pulmonary function tests (Fig. 2C, D). Follow-up flexible bronchoscopy also revealed that there was no regrowth of the neurofibroma, and the procedure site was healed with fibrotic scar formation with no other abnormalities (Fig. 2E). Up to 18 months after the completion of serial cryotherapy, the patient had no recurrent symptoms or complaints.

## 3. Discussion

Neurofibromas are usually associated with neurofibromatosis, especially type I. Isolated or solitary neurofibromas can occur without neurofibromatosis. The posterior mediastinum is the most common site of neurofibromas, and isolated endotracheal neurofibromas are rare pathologic conditions. Currently, the standard therapeutic option for neurofibromas causing clinically significant symptoms is surgery. For various airway pathologic conditions, interventional bronchoscopy has yielded clinical benefits as an alternative to surgery, thanks to the advancement of bronchoscopic technology. Recently, bronchoscopic cryotherapy has received attention from many physicians for its application to various types of endobronchial and endotracheal pathogenic conditions. Furthermore, the European Respiratory Society and American Thoracic Society guidelines recommend possible use of bronchoscopic cryotherapy for treatment of low-grade or noninvasive airway malignancies, foreign body removals, transbronchial biopsies, and endobronchial biopsies.^[6]^ In the current case, symptomatic endotracheal neurofibroma was treated by repeated flexible bronchoscopic cryotherapy.

Cryotherapy is a semi-invasive procedure with low risk of complications, although cryotherapy-associated complications include bleeding, cardiac arrhythmias, bronchospasm, and death.^[7]^ Cryotherapy yields clinical benefits by reducing pathologic tissues induced by rapid freezing of tissues, resulting in cell destruction and death. Therefore, bronchoscopic cryotherapy must be performed with caution to avoid accidental freezing injury of tissues adjacent to the target pathologic lesions. Tissues containing high water content are more sensitive to cryotherapy and are more easily damaged. The freezing-sensitive tissues include tumors, granulation tissue, skin, mucous membranes, nerves, and endothelium; fat, cartilage, nerve sheath, connective tissue, and fibrosis are relatively freezing-resistant due to their low water content.^[8]^

Given that neurofibroma is caused by proliferation of Schwann cells, fibroblasts, perineural cells, and others, neurofibroma appears to be less sensitive to cryotherapy. Very few case reports have shown the therapeutic efficacy of cryotherapy for neurofibromas in the central airway. In our current case, we performed repeated flexible bronchoscopic cryotherapies to remove an obstructing neurofibroma in the trachea. This strategy was appropriate to manage a tumor less that is sensitive to cryotherapy. The trachea is mostly composed of hyaline cartilage. Therefore, it may be hypothesized that the normal trachea structures were relatively tolerant and less likely to be damaged during cryotherapy. Based on these considerations, we could perform repeated bronchoscopic cryotherapy without any significant local complications such as tracheal stricture or perforation due to repetitive cryoinjuries of the adjacent normal tissues.

In conclusion, this case report is the first to highlight the therapeutic effects of repeated flexible bronchoscopic cryotherapy for a symptomatic endotracheal neurofibroma. Because there has been little information regarding the safety and efficacy of repeated bronchoscopic cryotherapy on central airway obstruction, it is worth noting that flexible bronchoscopic cryotherapy can be performed repeatedly within short durations for therapeutic purposes. This case report is expected to provide physicians with an alternative for patients with central airway obstruction due to tumorous lesions such as neurofibromas.

### Acknowledgments

This study was performed under a project entitled “Medical school students training program: Dream of being a Nobel Prize Winner,” part of the Research Center for Pulmonary Disorders at the Jeonbuk National University Medical School and Research Institute of Clinical Medicine of the Jeonbuk National University, Biomedical Research Institute at the Jeonbuk National University Hospital.

### Author contributions

All authors have read and approved the article, and significantly contributed to this article.

Conceptualization: Yong Chul Lee, So Ri Kim.

Data curation: Ji Soo Yoo, So Ri Kim.

Investigation: Yeong Hun Choe, Yong Chul Lee.

Writing – original draft: Ji Soo Yoo, So Ri Kim.

Writing – review & editing: So Ri Kim.

## References

[1] Peltonen J Jaakkola S Lebwohl M. Cellular differentiation and expression of matrix genes in type 1 neurofibromatosis. Lab Invest. 1989;59:760–71. 2462129

[2] Hsu HS Wang CY Li WY. Endotracheobronchial neurofibromas. Ann Thorac Surg. 2002;74:1704–6. 12440641 10.1016/s0003-4975(02)03958-9

[3] Scarlata S Fuso L Lucatoni G. The technique of endoscopic airway tumor treatment. J Thorac Dis. 2017;9:2619–39. 28932570 10.21037/jtd.2017.07.68 PMC5594167

[4] Tomic R Podgaetz E Andrade RS. Cryotechnology in diagnosing and treating lung diseases. J Bronchology Interv Pulmonol. 2015;22:76–84. 25590490 10.1097/LBR.0000000000000103

[5] Gage AA Koepf S Wehrle D. Cryotherapy for cancer of the and oral cavity. Cancer. 1965;l8:1646–51. 10.1002/1097-0142(196512)18:12<1646::aid-cncr2820181221>3.0.co;2-y 5845803

[6] DiBardino DM Lanfranco AR Hass AR. Bronchoscopic cryotherapy. Clinical applications of the cryoprobe, cryospray, and cryoadhesion. Ann Am Thorac Soc. 2016;13:1405–15. 27268274 10.1513/AnnalsATS.201601-062FR

[7] Du Rand IA Barber PV Goldring J. British Thoracic Society guideline for advanced diagnostic and therapeutic flexible bronchoscopy in adults. Thorax. 2011;66(Suppl 3):1–21. 21987439 10.1136/thoraxjnl-2011-200713

[8] Colella S Ravaglla C Tomassetti S. Cryotherapy: application in the airways. Díaz-Jimenez JP Rodrigues AN, eds. In: Interventions in Pulmonary Medicine. 2nd ed. Springer Cham, Switzerland 2018:139–153.

